# The chemoprotective effect of anti-platelet agents on cancer incidence in people with non-alcoholic fatty liver disease (NAFLD): a retrospective cohort study

**DOI:** 10.1186/s12916-024-03802-4

**Published:** 2024-12-03

**Authors:** Matthew Anson, Jun Shang Poon, Alex E. Henney, David Riley, Gema H. Ibarbaru, Cyril Sieberhagen, Daniel J. Cuthbertson, Uazman Alam, Theresa Hydes

**Affiliations:** 1 Department of Cardiovascular and Metabolic Medicine, Institute of Life Course and Medical Sciences, University of Liverpool, Liverpool, UK; 2 Liverpool Centre for Cardiovascular Science, University of Liverpool, Liverpool, UK; 3 University Hospital Aintree, Liverpool University Hospitals NHS Foundation Trust, Liverpool, UK; 4 TriNetX LLC, Cambridge, MA USA

**Keywords:** Hepatocellular carcinoma, Non-alcoholic fatty liver disease, Aspirin, Anti-platelets, Obesity

## Abstract

**Background:**

Non-alcoholic fatty liver disease (NAFLD) is associated with an increased incidence of hepatic and extrahepatic cancers, in particular those linked to obesity. In people with chronic liver disease, aspirin may confer protection against hepatocellular carcinoma (HCC). We explore the potential chemoprotective effect of aspirin/other anti-platelet agents on obesity-related cancers, including HCC in people with NAFLD.

**Methods:**

We performed a retrospective cohort study of anonymised electronic medical records using the TriNetX network (Cambridge, MA, USA), a global federated database. We identified adults aged 18 or over with a diagnosis of NAFLD, prior to commencing antiplatelet agents. Two groups were created: antiplatelet (1) versus no antiplatelet use (2). We propensity score matched for nine variables. Antiplatelet use was defined as aspirin, ticagrelor, cangrelor, clopidogrel or prasugrel use for at least 1 year. The outcomes of interest were incidence of HCC and other obesity-related cancers. Follow-up was for 5 years. We performed subgroup analyses on aspirin users only and stratified findings for sex and age. Sensitivity analysis was conducted on individuals with 3- and 5-year aspirin exposure.

**Results:**

Post matching, there were 42,192 people per group. Antiplatelet use in people with NAFLD was associated with statistically significant reduction in all obesity-related cancers (HR 0.71, 95% CI 0.65–0.78, *p* < 0.001) and individually for HCC (HR 0.52, 95% CI 0.40–0.68, *p* < 0.001), breast carcinoma (HR 0.78, 95% CI 0.66–0.92, *p* = 0.003), pancreatic carcinoma (HR 0.61, 95% CI 0.47–0.78, *p* < 0.001) and colorectal carcinoma (HR 0.68, 95% CI 0.56–0.84, *p* < 0.001). For women, there was a significant reduction in risk of ovarian carcinoma (HR 0.75, 95% CI 0.57–0.98, *p* = 0.034). Aspirin monotherapy was similarly associated with reduced incidence of HCC (HR 0.46, 95% CI 0.32–0.64, *p* < 0.001) and all obesity-related cancers (HR 0.71, 95% CI, 0.56–0.90, *p* = 0.004), with benefits observed in males (HR 0.71, 95% CI 0.56–0.90, *p* = 0.004), females (HR 0.77, 95% CI 0.67–0.88, *p* < 0.001) and in older (HR 0.72, 95% CI 0.63–0.82, *p* < 0.001) but not younger people (HR 0.78, 95% CI 0.60–1.03, *p* = 0.589).

**Conclusions:**

Aspirin/antiplatelet agents may have a role in primary cancer prevention in people living with NAFLD.

**Supplementary Information:**

The online version contains supplementary material available at 10.1186/s12916-024-03802-4.

## Background

Obesity is a chronic complex disease associated with a wide range of health complications including medical (cardiovascular disease, type 2 diabetes, non-alcoholic fatty liver disease (NAFLD)), musculoskeletal, mental health complications and risk of multiple malignancies (oesophageal, gastric, colorectal, liver, pancreatic, gall bladder, breast, uterine, ovarian, thyroid, meningioma, multiple myeloma) [1]. Prevalence of obesity has doubled from 1990 to 2022, according to World Health Organisation data, with 43% of adults living with been overweight or obese (60% in Europe and 67% in the Americas) [2].

NAFLD has emerged as the leading cause of chronic liver disease, affecting up to one third of the global population [3]. NAFLD occurs where there is excessive hepatic fat accumulation with secondary inflammation and potentially fibrosis. NAFLD is now the main driver for increased chronic liver disease incidence [4] and is associated with cirrhosis and hepatocellular carcinoma (HCC). As a multisystem disorder, it is also independently associated with cardiovascular disease [5], chronic kidney disease [6] and extra-hepatic cancer [7]. A recent meta-analysis including 10 cohort studies (182,202 people, median follow-up 5.8 years) identified that NAFLD was significantly associated with a 1.5- to 2.0-fold increased risk of incident gastrointestinal cancers (oesophagus, stomach, pancreas, colorectal cancers) and a 1.2- to 1.5-fold increased risk of lung, breast, gynaecological or urinary system [7]. These risks were independent of age, sex, smoking, obesity and diabetes status, although many patients will have multiple common metabolic-related oncogenic risk factors.

Obesity also may adversely affect/limit oncological treatment options, increase the risk of cancer-related mortality and increase rates of disease recurrence [8]. Prevention and early detection/timely treatment of cancer are therefore critical for people living with obesity. There has been increasing interest in the use of aspirin, an inhibitor of cyclooxygenase (COX)−2, for primary prevention of cancer and to improve cancer survival post diagnosis. Evidence is strongest for colorectal and other gastrointestinal tract [9]. Aspirin has also been shown to be protective against HCC in the general population and people with chronic liver disease in recent meta-analyses [10, 11], although no benefit was seen for people with cirrhosis who are at the highest risk of HCC [11]. For individuals with NAFLD, aspirin has recently been shown to halve the risk of HCC in a large retrospective study using Taiwan’s National Health Insurance Research Database (145,212 NAFLD patients, 33,484 received daily aspirin for 90 days or more and 55,543 patients did not receive any antiplatelet therapy, adjusted hazard ratio, HR 0.48, 95% CI 0.37–0.63) [12]. To our knowledge, the benefits of either aspirin or other anti-platelet agents has not been explored in people with NAFLD for prevalence of other cancers associated with obesity. This is highly clinically relevant given the high burden of metabolic related (oncogenic) risk factors in this population. Using a large international cohort of patients, we therefore explored the chemoprotective role of aspirin, and other anti-platelet agents, for the prevention of HCC and other cancers associated with obesity in the literature.

## Methods

### Network characteristics

We performed a retrospective cohort study using the TriNetX (TriNetX LLC, Cambridge, MA, USA) platform. The TriNetX research platform is a global collaborative network providing access to real-time anonymised electronic medical records. TriNetX has data usage and publication agreements in place with all health care organisations (HCOs). The TriNetX Global Collaborative network composes of over 135 million individuals across over 100 health care organisations (HCOs), primarily, secondary and tertiary units in North America and Europe. Data contained with the network includes demographics, diagnosis, procedures, medications and health care utilisation. We conformed to Strengthening the Reporting of Observational Studies in Epidemiology (STROBE) guidelines (Additional File 1: Table S1). The data used in this study was collected on 30 March 2024.

### Primary cohorts

We identified all adults, aged 18 or over, with NAFLD, defined by the presence of International Classification of Diseases 10th revision (ICD-10) codes K75.8 (other specified inflammatory liver diseases) or K76.0 (fatty change of the liver not elsewhere classified). We excluded individuals with other causes of chronic liver disease (Additional File 1: Table S2).

**Fig. 1:**
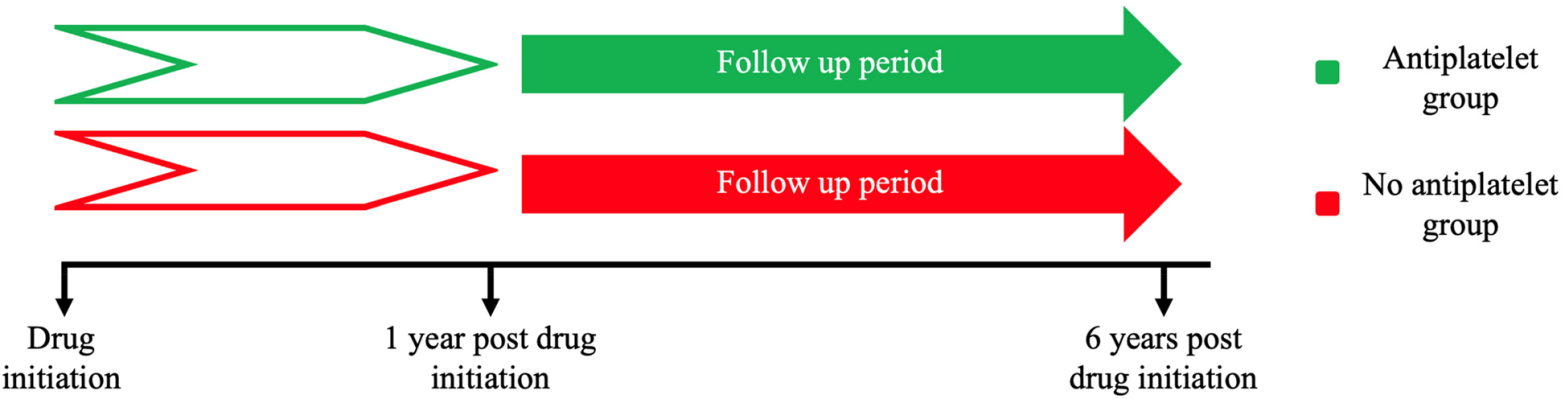
Timeline of included individuals for the main cohort analysis

Two groups were created: (1) anti-platelet use and (2) no anti-platelet use. Anti-platelet use was defined as aspirin, ticagrelor, cangrelor, clopidogrel or prasugrel use for at least 1 year. Individuals must have had a diagnosis of NAFLD prior to commencement of antiplatelet use. We adopted an active comparator new user design where analysis was of new starters of antiplatelets. Individuals in the ‘no antiplatelet use’ group must not have had any coding of antiplatelet use in their electronic medical record. The index event for antiplatelet users was defined at 1 year post initiation of medication, and the index event for non-users was defined at 1 year post the first diagnosis of NAFLD (Fig. 1). In addition, we performed an analysis of people using aspirin monotherapy as compared to people not prescribed any anti-platelet agents.

Groups were propensity score matched (PSM) for age at index event, sex, ethnicity, presence of type 2 diabetes (T2D) (ICD-10 E11), obesity (defined as BMI ≥ 30 kg/m^2^), all neoplasms (ICD-10 C00-D49), ischaemic heart diseases (ICD-10 I20-25), cerebrovascular disease (ICD-10 I60-69) and other peripheral vascular disease (ICD-10 I73). Primary outcome was incidence of new HCC [13] (ICD-10 C22). Secondary outcomes included incidence of other cancers known to be associated with obesity according to the literature (oesophageal [14], gastric [15], colorectal [16], gallbladder [17], pancreatic [18], breast [19], ovarian [20], uterine [21], thyroid [22], multiple myeloma [23]) (ICD codes in Additional File 1: Table S3). Individuals with a history of an outcome of interest were excluded from prospective analysis of that respective outcome only. Individuals were followed up for 5 years post index event. Participants that died during the study period or who were lost to follow-up (e.g. moving to another HCO not included within the network) were censored at that time point. Sensitivity analysis was conducted for 3- and 5-year aspirin exposure, with outcomes analysed from the point of drug initiation.

### Statistical analysis

Due to the nature of the data source, this dataset may face some typical data quality challenges of EMRs such as incomplete or inaccurate data entries, under-reporting of certain conditions, limited granularity and exclusion of data not integrated into the HCO’s EMR. Nevertheless, TriNetX employs data validation processes to ensure the accuracy and reliability of its data. These processes include regular data quality checks to identify and correct discrepancies, validation against external benchmarks to ensure consistency and accuracy and collaboration with data contributors to resolve any identified issues and improve data quality continuously. Statistical analysis is conducted within the TriNetX platform using the R survival package as a backbone. Groups were 1:1 propensity score matched (PSM). We used greedy nearest neighbour matching with a caliper of 0.1 pooled standard deviations. The proportional hazard assumption was tested using the generalised Schoenfeld approach built into the platform. Hazard ratios alongside 95% CI and *p* values are reported for the prospective analysis and mean and standard deviation for baseline characteristics. Kaplan–Meier curves were calculated for survival probability. If the last data entry (outcomes of interest, date of death, end of data collection, or loss to follow-up) in the patient’s record was in the time window for analysis, the patient was censored on the day after the last fact in their record. We calculated the *E*-value for each outcome of interest. The *E*-value is defined as the minimum strength of association, on the risk ratio scale that an unmeasured confounder would need to have with both treatment and outcome to fully explain away a specific treatment-outcome association [24]. There is no threshold of significance for the *E*-value and should be interpreted in context with the size of the HR [25]. Variables with strictly standardised mean difference (SSMD) < 0.1 is well matched between groups. Statistical significance is set at the 5% level.

## Results

### Baseline characteristics

We identified a total of 1,086,684 individuals. 42,649 had been issued an antiplatelet agent for at least 1 year post diagnosis of NAFLD, and 1,044,035 had no coding of any anti-platelet agent use ever. Post PSM, there were 42,192 in each group. Aspirin monotherapy was the most frequent group consisting of 66.9% of all individuals (*n* = 28,220). Clopidogrel, prasugrel, ticagrelor and cangrelor monotherapy made up 2.6%, 0.5%, 0.4% and < 0.1% respectively. The remaining 29.0% of individuals used a combination of antiplatelet therapy. For individuals prescribed ‘any’ antiplatelet, 93% included a prescription for aspirin. Table 1 summarises the baseline demographics.

**Table 1:** Baseline patient demographics and characteristics post propensity score matching

	**Anti-platelet use (*n* = 42,192)**	**No antiplatelet use (*n* = 42,192)**	**Strictly standardised mean difference**
**Demographics**			
Age at index event (years)	62.8 ± 12.6	63.2 ± 12.7	0.029
Sex (female) [%]	58	57	0.007
Race (White/Black or African American/Asian) [%]	71/9/4	70/9/3	0.011/0.006/0.012
**Anthropometrics**			
Body mass index (kg/m^2^)	33.4 ± 7.6	33.9 ± 7.5	0.062
**Comorbidities** [%]			
Type 2 diabetes	33.8	33.4	0.009
Ischaemic heart disease	20.1	19.8	0.008
History of any neoplasm	14.4	14.8	0.012
Cerebrovascular disease	6.7	6.4	0.010
Other peripheral vascular disease	3.3	3.0	0.019

### Incidence of obesity-related cancer according to any anti-platelet use for people with NAFLD

#### Effect of any anti-platelet use on cancer incidence for people with NAFLD

**Fig. 2:**
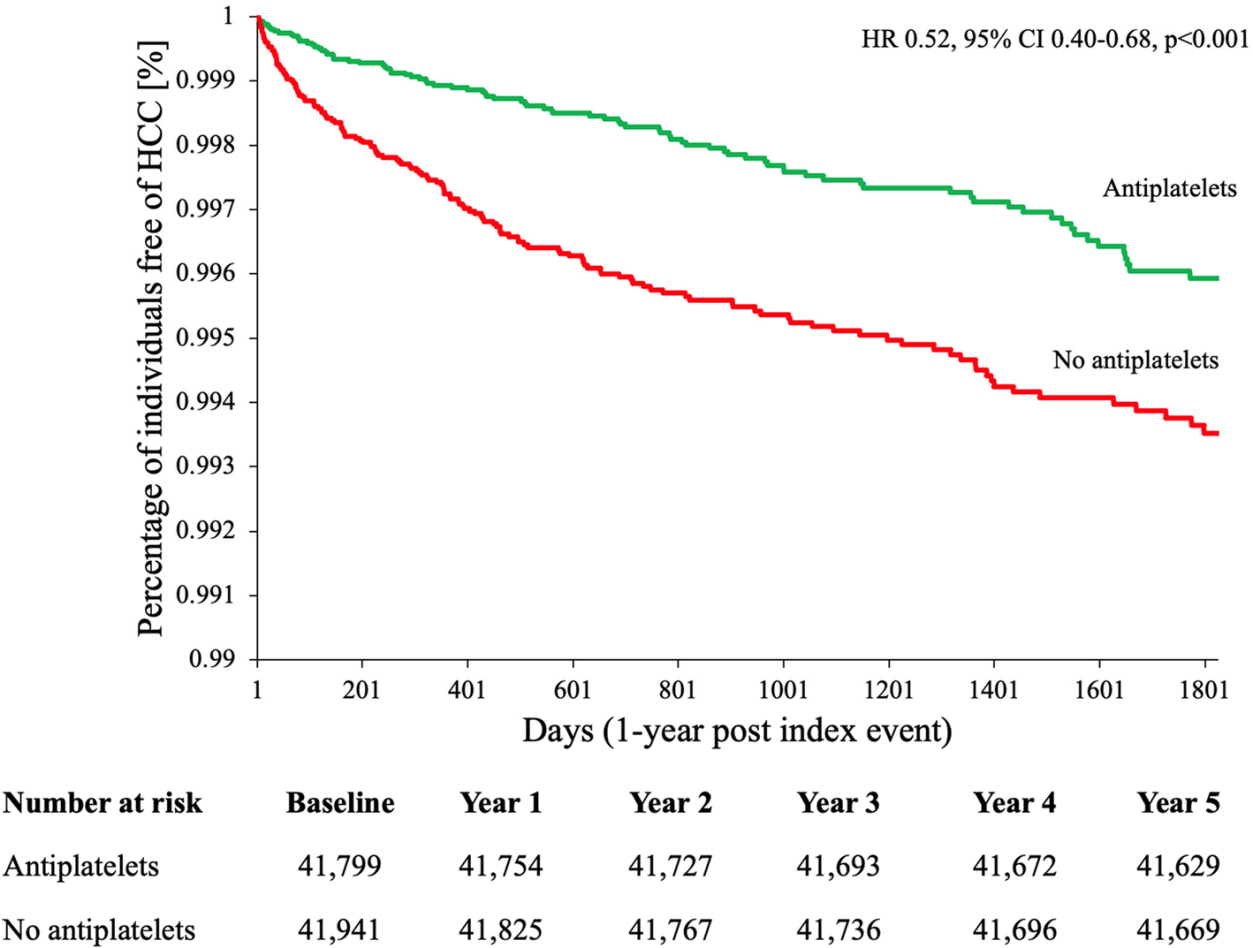
Kaplan–Meier estimates of time to HCC diagnosis

Overall, there were 796 cancer events in people prescribed anti-platelets and 1033 events for people not prescribed anti-platelets respectively. Antiplatelet use was associated with statistically significant reduction in all obesity-related cancers (HR 0.71, 95% CI 0.65–0.78, *p* < 0.001, *E*-value 1.85) in people with NAFLD (Table 2). Antiplatelet use was associated with a significantly reduced risk of HCC (HR 0.52, 95% CI 0.40–0.68, *p* < 0.001, *E*-value 2.52) compared to non-users (Fig. 2). Benefit was observed after a year of prescription and increased over time (Fig. 2).

**Table 2:** Summary of outcomes stratified for all antiplatelet and aspirin only users

	**All antiplatelets (*n* = 84,304)**					**Aspirin only (*n* = 56,440)**				
	**Sample size**	**Outcome (*n*)**	**HR (95% confidence interval)**	***p* value**	***E* value**	**Sample size**	**Outcome (*n*)**	**HR (95% confidence interval)**	***p* value**	***E* value**
***All obesity-related cancers***^**a**^										
Antiplatelet(s)	37,757	796	**0.71 (0.65–0.78)**	** < 0.001**	**1.85**	15,417	280	**0.64 (0.55–0.74)**	** < 0.001**	**2.06**
No antiplatelets	38,536	1033				15,421	407			
***Hepatocellular carcinoma***										
Antiplatelet(s)	41,799	89	**0.52 (0.40–0.68)**	** < 0.001**	**2.52**	27,953	48	**0.46 (0.32–0.64)**	** < 0.001**	**2.80**
No antiplatelets	41,941	156				28,061	99			
***Colorectal carcinoma***										
Antiplatelet(s)	41,292	162	**0.68 (0.56–0.84)**	** < 0.001**	**1.94**	27,564	93	**0.63 (0.49–0.82)**	**0.001**	**2.10**
No antiplatelets	41,406	217				27,710	138			
***Pancreatic carcinoma***										
Antiplatelet(s)	41,963	101	**0.61 (0.47–0.78)**	** < 0.001**	**2.16**	28,054	63	**0.61 (0.45–0.84)**	**0.002**	**2.16**
No antiplatelets	41,883	152				27,997	96			
***Oesophageal carcinoma***										
Antiplatelet(s)	41,119	26	0.70 (0.42–1.17)	0.174	1.00	28,169	16	0.58 (0.31–1.08)	0.082	2.27
No antiplatelets	41,093	34				28,150	26			
***Gastric carcinoma***										
Antiplatelet(s)	42,079	43	0.76 (0.50–1.13)	0.171	1.00	28,144	29	0.73 (0.45–1.19)	0.207	1.00
No antiplatelets	42,075	52				28,137	37			
***Gallbladder carcinoma***										
Antiplatelet(s)	42,162	10^b^	0.66 (0.29–1.48)	0.305	1.00	28,197	10^b^	0.62 (0.22–1.75)	0.365	1.00
No antiplatelets	42,156	14				28,195	10^b^			
***Ovarian carcinoma***										
Antiplatelet(s)	41,979	58	0.87 (0.61–1.25)	0.443	1.00	28,061	41	0.85 (0.56–1.30)	0.457	1.00
No antiplatelets	42,004	61				28,089	45			
***Uterine carcinoma***										
Antiplatelet(s)	41,718	93	**0.75 (0.57–0.98)**	**0.034**	**1.74**	27,881	71	0.81 (0.59–1.11)	0.191	1.00
No antiplatelets	41,751	114				27,877	82			
***Breast carcinoma***										
Antiplatelet(s)	17,535	255	**0.78 (0.66–0.92)**	**0.003**	**1.66**	26,885	173	**0.80 (0.65–0.97)**	**0.024**	**1.61**
No antiplatelets	17,502	302				27,236	206			
***Multiple myeloma***										
Antiplatelet(s)	41,910	63	0.98 (0.68–1.39)	0.889	1.00	28,007	36	0.79 (0.50–1.22)	0.283	1.00
No antiplatelets	42,051	59				28,146	43			
***Thyroid carcinoma***										
Antiplatelet(s)	41,802	71	0.82 (0.60–1.13)	0.225	1.00	27,933	48	0.92 (0.62–1.36)	0.665	1.00
No antiplatelets	41,949	79				28,044	49			

^a^Individuals were censored at the first coding of a constituent obesity-related malignancy composite outcome. The total number of individuals experiencing the composite outcome differ than that of the sum of the individual events because, to better ascertain the primary preventative effect of aspirin on all obesity-related malignancies, individuals with a history of any of the constituent events were excluded from analysis of the composite outcome

^b^TriNetX implements several safeguards to minimise the risk of patient reidentification. To avoid the risk that a series of individual queries could identify small subsets of cohorts, when a query returns a patient count on an outcome where the patient count is ≤ 10 but greater than 0, the count is obfuscated to 10. The reported HR is calculated without this obfuscation present

Antiplatelet agent use was associated with statistically significant reduction in incident breast carcinoma (HR 0.78, 95% CI 0.66–0.92, *p* = 0.003, *E*-value 1.66), pancreatic carcinoma (HR 0.61, 95% CI 0.47–0.78, *p* < 0.001, *E*-value 2.16) and colorectal carcinoma (HR 0.68, 95% CI 0.56–0.84, *p* < 0.001, *E*-value 1.94). For women, there was a significant reduction in risk of uterine carcinoma (HR 0.75, 95% CI 0.57–0.98, *p* = 0.034, *E*-value 1.74) (Table 2). There was no significant difference between users and non-users of anti-platelet agents in incidence of gallbladder carcinoma (HR 0.66, 95% CI 0.29–1.48, *p* = 0.305), gastric carcinoma (HR 0.76, 95% CI 0.50–1.13, *p* = 0.171), oesophageal carcinoma (HR 0.70, 95% CI 0.42–1.17, *p* = 0.174), ovarian carcinoma (HR 0.87, 95% CI 0.61–1.25, *p* = 0.443), multiple myeloma (HR 0.98, 95% CI 0.68–1.39, *p* = 0.889) and thyroid carcinoma (HR 0.82, 95% CI 0.60–1.13, *p* = 0.225) (Table 2).

#### Effect of any anti-platelet use on cancer incidence for people with NAFLD stratified by sex

**Fig. 3:**
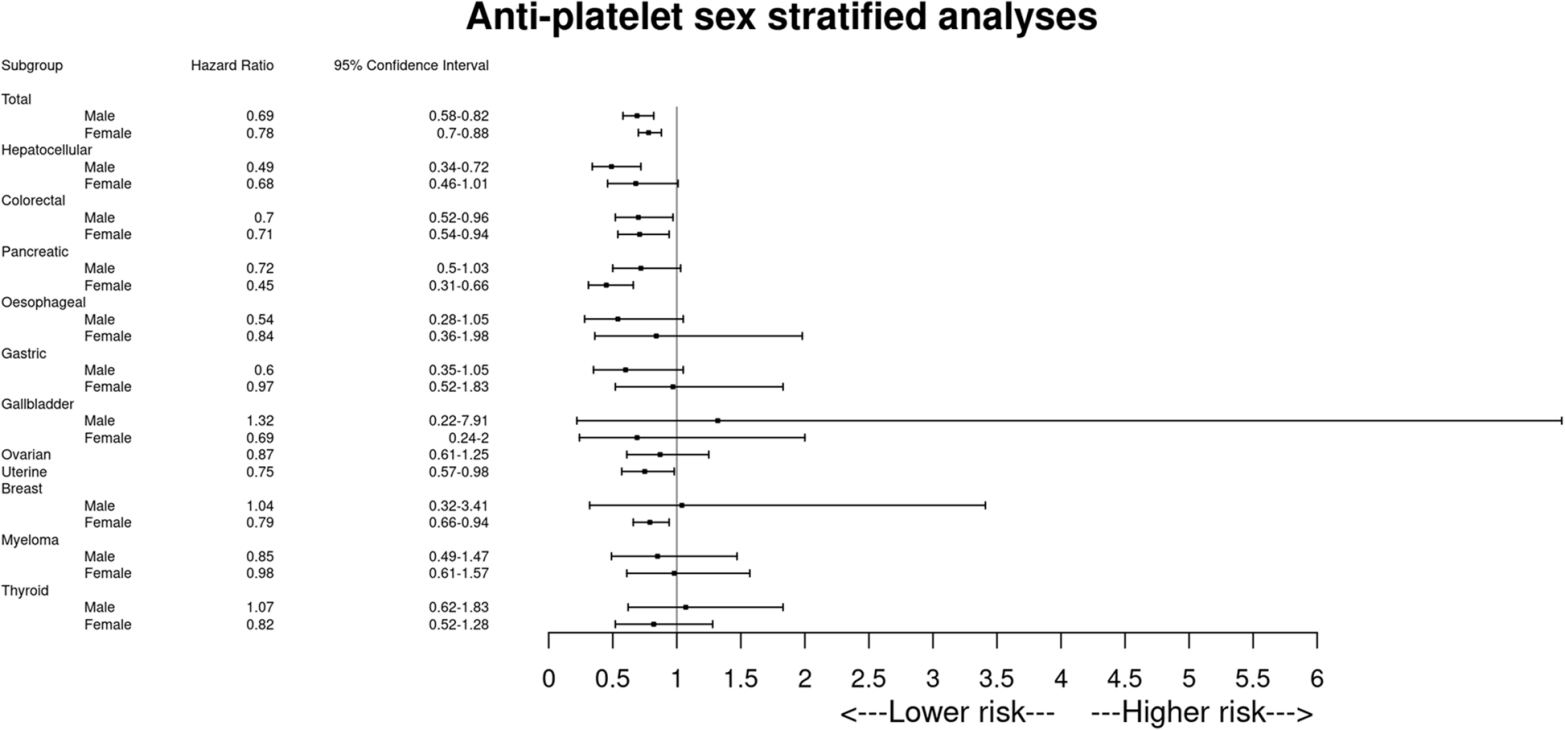
Forest plot of all clinical outcomes at 5 years in all antiplatelet users with NAFLD, sub-stratified by sex

A reduced incidence of all obesity-related cancer according to anti-platelet use was observed for both men (HR 0.69, 95% CI 0.58–0.82, *p* < 0.001, *E*-value 1.91) and women (HR 0.78, 95% CI 0.70–0.88, *p* < 0.001, *E*-value 1.66) (Table 3, Fig. 3). For men, antiplatelet use was associated with reduced risk of HCC (HR 0.49, 95% CI 0.34–0.72, *p* < 0.001, *E*-value 2.66) and colorectal carcinoma (HR 0.70, 95% CI 0.52–0.96, *p* = 0.026, *E*-value 1.88). For women, a benefit was observed for colorectal carcinoma (HR 0.71, 95% CI 0.54–0.94, *p* = 0.018, *E*-value 1.85), pancreatic carcinoma (HR 0.45, 95% CI 0.31–0663, *p* < 0.001, *E*-value 2.86) and uterine carcinoma as described previously (Table 3, Fig. 3).

**Table 3:** Summary of outcomes stratified by sex for all antiplatelet users

	**Male (*n* = 35,630)**					**Female (*n* = 44,240)**				
	**Sample size**	**Outcome (*n*)**	**HR (95% confidence interval)**	***p* value**	***E* value**	**Sample size**	**Outcome (*n*)**	**HR (95% confidence interval)**	***p* value**	***E* value**
***All obesity-related cancers***^**a**^										
Antiplatelets	16,749	224	**0.69 (0.58–0.82)**	** < 0.001**	**1.91**	19,003	525	**0.78 (0.70–0.88)**	** < 0.001**	**1.66**
No antiplatelets	16,937	293				19,639	638			
***Hepatocellular carcinoma***										
Antiplatelets	17,589	43	**0.49 (0.34–0.72)**	** < 0.001**	**2.66**	21,967	44	0.68 (0.46–1.01)	0.053	1.00
No antiplatelets	17,667	78				22,035	60			
***Colorectal carcinoma***										
Antiplatelets	17,391	71	**0.70 (0.52–0.96)**	**0.026**	**1.88**	21,691	85	**0.71 (0.54–0.94)**	**0.018**	**1.85**
No antiplatelets	17,420	89				21,779	111			
***Pancreatic carcinoma***										
Antiplatelets	17,707	54	0.72 (0.50–1.03)	0.070	1.00	22,012	39	**0.45 (0.31–0.66)**	** < 0.001**	**2.86**
No antiplatelets	17,693	67				21,963	81			
***Oesophageal carcinoma***										
Antiplatelets	17,768	14	0.54 (0.28–1.05)	0.066	1.00	22,096	10^b^	0.84 (0.36–1.98)	0.690	1.00
No antiplatelets	17,760	23				22,106	11			
***Gastric carcinoma***										
Antiplatelets	17,760	21	0.60 (0.35–1.05)	0.068	1.00	22,070	20	0.97 (0.52–1.83)	0.935	1.00
No antiplatelets	17,759	31				22,081	19			
***Gallbladder carcinoma***										
Antiplatelets	17,787	10^b^	1.32 (0.22–7.91)	0.759	1.00	22,109	10^b^	0.69 (0.24–2.00)	0.494	1.00
No antiplatelets	17,802	10^b^				22,098	10^b^			
***Ovarian carcinoma***										
Antiplatelets	N/A					21,921	58	0.87 (0.61–1.25)	0.443	1.00
No antiplatelets						21,932	61			
***Uterine carcinoma***										
Antiplatelets	N/A					21,685	93	**0.75 (0.57–0.98)**	**0.034**	**1.74**
No antiplatelets						21,715	114			
***Breast carcinoma***										
Antiplatelets	17,783	10^b^	1.04 (0.32–3.41)	0.947	1.00	20,448	235	**0.79 (0.66–0.94)**	**0.006**	**1.63**
No antiplatelets	17,791	10^b^				20,878	281			
***Multiple myeloma***										
Antiplatelets	17,689	25	0.85 (0.49–1.47)	0.553	1.00	10,755	36	0.98 (0.61–1.57)	0.934	1.00
No antiplatelets	17,754	26				10,799	34			
***Thyroid carcinoma***										
Antiplatelets	17,714	29	1.07 (0.62–1.83)	0.819	1.00	21,852	36	0.82 (0.52–1.28)	0.369	1.00
No antiplatelets	17,756	24				21,975	41			

^a^Individuals were censored at the first coding of a constituent obesity-related malignancy composite outcome. The total number of individuals experiencing the composite outcome differ than that of the sum of the individual events because, to better ascertain the primary preventative effect of aspirin on all obesity-related malignancies, individuals with a history of any of the constituent events were excluded from analysis of the composite outcome

^b^TriNetX implements several safeguards to minimise the risk of patient reidentification. To avoid the risk that a series of individual queries could identify small subsets of cohorts, when a query returns a patient count on an outcome where the patient count is ≤ 10 but greater than 0, the count is obfuscated to 10. The reported HR is calculated without this obfuscation present

### Incidence of obesity-related cancer according to aspirin monotherapy use for people with NAFLD

#### Effect of aspirin monotherapy use on cancer incidence for people with NAFLD

Overall, 28,220 individuals were issued aspirin only post PSM. Aspirin monotherapy was associated with statistically significant reduction in incident obesity-related cancers combined (HR 0.64, 95% CI 0.55–0.74, *p* < 0.001, *E*-value 2.06). For individual cancers, aspirin use was found to be protective for HCC (HR 0.46, 95% CI 0.32–0.64, *p* < 0.001, *E*-value 2.80), colorectal carcinoma (HR 0.63, 95% CI 0.49–0.82, *p* = 0.001, *E*-value 2.10), pancreatic carcinoma (HR 0.61, 95% CI 0.45–0.84, *p* = 0.002, *E*-value 2.16) and breast carcinoma (HR 0.80, 95% CI 0.65–0.97, *p* = 0.024, *E*-value 1.61). There was no significant difference between aspirin users and non-users of anti-platelets in incidence of oesophageal carcinoma (HR 0.58, 95% CI 0.31–1.08, *p* = 0.082), gastric carcinoma (HR 0.73, 95% CI 0.45–1.19, *p* = 0.207), gallbladder carcinoma (HR 0.62, 95% CI 0.22–1.75, *p* = 0.365), multiple myeloma (HR 0.79, 95% CI 0.50–1.22, *p* = 0.283) or thyroid carcinoma (HR 0.92, 95% CI 0.62–1.36, *p* = 0.665) (Table 2).

#### Effect of aspirin monotherapy use on cancer incidence for people with NAFLD stratified by sex

**Fig. 4:**
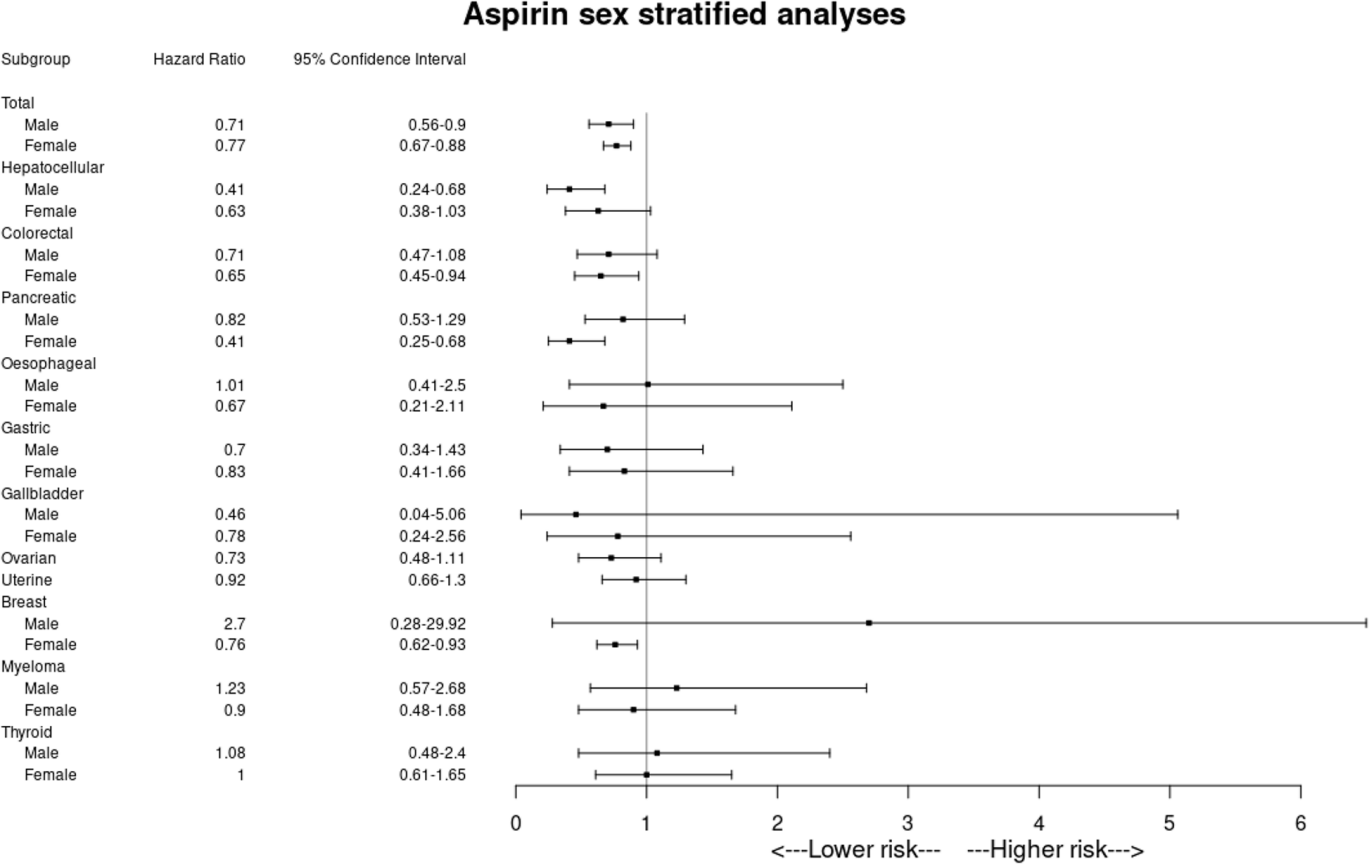
Forest plot of all clinical outcomes at 5 years in aspirin monotherapy users with NAFLD, sub-stratified by sex

For women, aspirin prescription was associated with reduced incidence of all obesity-related cancers (HR 0.77, 95% CI 0.67–0.88, *p* < 0.001, *E*-value 1.69). Specifically, a significant risk reduction was observed for colorectal carcinoma (HR 0.65, 95% CI 0.45–0.94, *p* = 0.019, *E*-value 2.03), pancreatic cancer (HR 0.41, 95% CI 0.25–0.68, *p* < 0.001, *E*-value 3.09) and breast carcinoma (HR 0.76, 95% CI 0.62–0.93, *p* = 0.009, *E*-value 1.71). No statistically significant association was observed for other obesity-related cancers (Table 4, Fig. 4). For men, aspirin use was associated with reduced incidence of all obesity-related cancers (HR 0.71, 95% CI 0.56–0.90, *p* = 0.004, *E*-value 1.85) and HCC (HR 0.41, 95% CI 0.24–0.68, *p* < 0.001, *E*-value 3.09), (Table 4, Fig. 4).

**Table 4:** Summary of outcomes of aspirin users stratified by sex (people prescribed aspirin monotherapy vs non-users of any antiplatelets)

	**Male (*n* = 21,964)**					**Female (*n* = 31,584)**				
	**Sample size**	**Outcome (*n*)**	**HR (95% confidence interval)**	***p* value**	***E* value**	**Sample size**	**Outcome (*n*)**	**HR (95% confidence interval)**	***p* value**	***E* value**
***All obesity-related cancers***^**a**^										
Aspirin	10,271	125	**0.71 (0.56–0.90)**	**0.004**	**1.85**	13,469	346	**0.77 (0.67–0.88)**	** < 0.001**	**1.69**
No antiplatelets	10,441	162				13,999	437			
***Hepatocellular carcinoma***										
Aspirin	10,846	21	**0.41 (0.24–0.68)**	** < 0.001**	**3.09**	15,671	26	0.63 (0.38–1.03)	0.064	1.00
No antiplatelets	10,888	47				15,739	39			
***Colorectal carcinoma***										
Aspirin	10,690	39	0.71 (0.47–1.08)	0.103	1.00	15,463	49	**0.65 (0.45–0.94)**	**0.019**	**2.03**
No antiplatelets	10,738	50				15,546	71			
***Pancreatic carcinoma***										
Aspirin	10,909	36	0.82 (0.53–1.29)	0.397	1.00	15,709	22	**0.41 (0.25–0.68)**	** < 0.001**	**3.09**
No antiplatelets	10,902	40				15,678	50			
***Oesophageal carcinoma***										
Aspirin	10,949	10^b^	1.01 (0.41–2.50)	0.979	1.00	15,776	10^b^	0.67 (0.21–2.11)	0.488	1.00
No antiplatelets	10,947	10^b^				15,780	10^b^			
***Gastric carcinoma***										
Aspirin	10,604	13	0.70 (0.34–1.43)	0.321	1.00	15,754	15	0.83 (0.41–1.66)	0.595	1.00
No antiplatelets	10,593	17				15,769	17			
***Gallbladder carcinoma***										
Aspirin	10,969	10^b^	0.46 (0.04–5.06)	0.514	1.00	15,783	10^b^	0.78 (0.24–2.56)	0.681	1.00
No antiplatelets	10,973	10^b^				15,775	10^b^			
***Ovarian carcinoma***										
Aspirin	N/A					15,643	38	0.73 (0.48–1.11)	0.140	1.00
No antiplatelets						15,647	49			
***Uterine carcinoma***										
Aspirin	N/A					15,473	66	0.92 (0.66–1.30)	0.642	1.00
No antiplatelets						15,475	67			
***Breast carcinoma***										
Aspirin	10,959	10^b^	2.70 (0.28–29.92)	0.371	1.00	14,558	160	**0.76 (0.62–0.93)**	**0.009**	**1.71**
No antiplatelets	10,970	10^b^				14,915	202			
***Multiple myeloma***										
Aspirin	10,892	15	1.23 (0.57–2.68)	0.603	1.00	15,676	19	0.90 (0.48–1.68)	0.731	1.00
No antiplatelets	10,943	11				15,759	20			
***Thyroid carcinoma***										
Aspirin	10,920	13	1.08 (0.48–2.40)	0.859	1.00	15,584	32	1.00 (0.61–1.65)	0.988	1.00
No antiplatelets	10,953	11				15,682	30			

^a^Individuals were censored at the first coding of a constituent obesity-related malignancy composite outcome. The total number of individuals experiencing the composite outcome differ than that of the sum of the individual events because, to better ascertain the primary preventative effect of aspirin on all obesity-related malignancies, individuals with a history of any of the constituent events were excluded from analysis of the composite outcome

^b^TriNetX implements several safeguards to minimise the risk of patient reidentification. To avoid the risk that a series of individual queries could identify small subsets of cohorts, when a query returns a patient count on an outcome where the patient count is ≤ 10 but greater than 0, the count is obfuscated to 10. The reported HR is calculated without this obfuscation present

#### Effect of aspirin monotherapy use on cancer incidence for people with NAFLD stratified by age

For individuals aged over 60, aspirin use was associated with reduced incidence of all obesity-related cancers (HR 0.72, 95% CI 0.63–0.82, *p* < 0.001, *E*-value 1.82), HCC (HR 0.41, 95% CI 0.28–0.59, *p* < 0.001, *E*-value 3.09), colorectal carcinoma (HR 0.67, 95% CI 0.51–0.90, *p* = 0.006, *E*-value 1.97), pancreatic carcinoma (HR 0.70, 95% CI 0.49–0.99, *p* = 0.040, *E*-value 1.88), gastric carcinoma (HR 0.59, 95% CI 0.36–0.97, *p* = 0.035, *E*-value 2.24) and breast carcinoma (HR 0.74, 95% CI 0.59–0.92, *p* = 0.005, *E*-value 1.77). Cancer incidence was significantly less in individuals aged 60 or less and aspirin was not associated with any reduced incidence of cancers (Additional File 1: Table S4, Additional File 1: Fig. S1).

#### Effect of aspirin monotherapy use on cancer incidence for people with NAFLD stratified by exposure time

Aspirin exposure for at least 3 and 5 years was associated with a statistically significant reduced incidence of all obesity-related cancers, HCC and pancreatic carcinoma (Additional File 1: Table S5, Additional File 1: Fig. S2).

#### Sensitivity analysis

Aspirin exposure for 3 years, with follow-up beginning from the point of drug initiation, was associated with a statistically significant reduced incidence of obesity-related cancers (HR 0.79, 95% CI 0.69–0.89, *p* < 0.001, *E*-value 1.63) and HCC (HR 0.55, 95% CI 0.39–0.78, *p* < 0.001, *E*-value 2.39). Additionally, aspirin exposure for 5 years, from the point of initiation, was associated with a greater reduced incidence of obesity-related cancers (HR 0.74, 95% CI 0.61–0.89, *p* = 0.001, *E*-value 1.77) and HCC (HR 0.44, 95% CI 0.26–0.76, *p* = 0.003, *E*-value 2.91).

## Discussion

Using a large global federation health research network, we demonstrate that use of anti-platelet agents (for which over 90% included a prescription for aspirin) is associated with reduced incidence of HCC and other cancers known to be linked to obesity, namely colorectal, breast, pancreatic and uterine cancer in people with NAFLD following propensity score matching for confounders. For aspirin monotherapy, there was a reduction in risk for HCC, colorectal, breast cancer and pancreatic cancer. This protection was lost for people under 60 years where cancer incidence was reduced. This is the first study in a Western population to examine the association between aspirin and incident HCC in people with NAFLD and the first to analyse the wider chemoprotective role of aspirin for other obesity-related cancers in this population.

Observational data supports a chemoprotective role of aspirin for HCC. In a recent systematic review and meta-analysis, aspirin use was associated with a lower incidence of HCC in a dose-dependent and duration-dependent manner and was also found to be associated with reduced recurrence and mortality [26]. In a further meta-analysis (12 cohort studies, 4 case–control studies; 822,680 aspirin users, 20,626 HCC cases), Abdelmalak et al. demonstrated that aspirin use reduces incident HCC by 30%, although this protection was not demonstrated for people with cirrhosis, a leading risk factor for HCC [11]. This association has not been studied in the setting of a randomised control trial (RCT) however. Few studies have examined a chemoprotective role for aspirin in people with NAFLD. This is of particular interest as the prevalence of NAFLD-associated HCC [27]. Lee et al. conducted a large retrospective cohort study to assess the relationship between aspirin and reduction in risk of HCC in people with NAFLD using Taiwan’s National Health Insurance Research database, involving 145,212 NAFLD patients, 33,484 patients continuously receiving a daily dose of aspirin for at least 90 days and 55,543 patients who did not receive any antiplatelet therapy [12]. Aspirin therapy was associated with a reduced HCC risk (adjusted HR 0.48 [95% CI 0.37–0.63]), and aspirin use for greater than 3 years was associated with the lowest risk of HCC. In this study, we observe a similar reduction in risk for both aspirin monotherapy and any platelet use for people with NAFLD. Following stratification for sex, protection against incident HCC in this study was only observed for men, which may be related to the higher incidence of primary liver cancer in this group. A sensitivity analysis, with follow-up starting from the point of antiplatelet initiation, demonstrated a reduction in incidence of obesity-related cancers and HCC, with a greater effect seen with 5-year aspirin exposure compared to 3 years.

We also demonstrate a reduction in incident colorectal cancer in addition to breast, uterine and pancreatic cancer for women and colorectal cancer for men for people with NAFLD prescribed any anti-platelet therapy. While observational data supports a protective role of aspirin use for colorectal cancer [28], meta-analysis of RCT data has shown conflicting results. Ma et al. concluded that while aspirin use overall did not reduce colorectal cancer incidence, a pooled analysis of studies which used low dose aspirin showed moderate benefit (relative risk 0.84) [29]. Aspirin was also found to reduce recurrence and cancer-related mortality [29]. Conversely, Shah et al. concluded that only high-dose aspirin is protective [30]. Ghaddaf et al. reported that aspirin use only reduces the risk of advanced lesions for up to 5 years [31]. Indeed, an updated evidence report and systemic review for the US Preventative Services Task Force (USPSTF) concluded that while low-dose aspirin was associated with small absolute risk reductions in major cardiovascular disease, ‘colorectal cancer results were less robust and highly variable’ [32].

With respect to the role of aspirin and incidence of other cancers, most data available for analysis is observational. Meta-analysis data do suggest a chemo-preventative benefit for aspirin for incident pancreatic cancer, in particular for people taking high-dose aspirin with a longer duration of use [33, 34]. Similarly, meta-analyses have reported a modest reduced incidence of gastric cancer (33 studies, risk ratio 0.89) [35] and breast cancer (42 studies, relative risk 0.92) [36], although there was significant heterogeneity between studies. Pooled analyses of 12 observational studies have identified a 13% reduction in incident ovarian cancer in all subgroups other than women with endometriosis [37]. This benefit is not borne out in RCT data however. In the Women’s Health Study, a randomised 2 × 2 factorial trial of aspirin 100 mg daily and aspirin placebo (39,876 US women) for 10 years did not reveal any difference in incidental cancer at any site other than non-melanoma skin cancer [38]. While other studies have identified reduced incidence of oesophageal cancer (metanalysis, 9 studies) [39], this data is observational only. In common with this study, no benefit has been demonstrated for aspirin against incidence myeloma [40] and minimal data exists for gallbladder cancer.

In this cohort, we identify that any anti-platelet use is associated with reduced incidence of HCC, colorectal cancer, pancreatic cancer, uterine cancer and breast cancer, and aspirin monotherapy was only found to be protective against all the above but not uterine cancer. It is unclear whether this is related to a lower number of people at risk and lower number of events in the aspirin monotherapy group or a compound effect of dual or consecutive anti-platelet use. Aspirin inhibits cyclooxygenase-2, which promotes inflammation and cell proliferation and inhibits nuclear factor kappa light chain induction of apoptosis. In terms of HCC, aspirin may influence carcinogenesis via reduction in hepatic fat content, recently demonstrated in a preliminary phase 2 trial [41]. Antiplatelet therapy (aspirin/clopidogrel) has also been shown to reduce intrahepatic platelet accumulation and platelet–immune cell interaction, limiting hepatic immune cell trafficking leading to attenuated intrahepatic cytokine and chemokine release, macrovesicular steatosis and hepatic inflammation [42].

The clinical implications of our findings are hugely significant considering NAFLD represents a major public health challenge [3]. Breast and colorectal carcinoma represent two of the leading causes of cancer in the UK. Although HCC is less common, survival rates are poor at it is the only cancer for which incidence and mortality rates are increasing [43]. Our study supports previous findings from observational research that anti-platelet therapy may be beneficial for primary prevention of common obesity-related cancers. However, these findings have not yet been convincingly reproduced in RCTs, the level of evidence required for clinical recommendations. In 2016, the US Preventive Services Task force (USPSTF) endorsed for the first time low-dose aspirin for prevention of colorectal cancer, in addition to cardiovascular disease, for individuals aged 50–59 years with a 10% 10-year cardiovascular risk [44]. However, in 2022, the USPSTF revised its recommendations regarding aspirin for primary prevention of CVD and withdrew its recommendation regarding colorectal cancer, citing ‘inadequate’ evidence to support aspirin’s reduction of colorectal cancer risk [32]. Therefore, future proof-of-concept trials should be performed to confirm, or refute, our findings, to help inform clinical guidelines. Such clinical guidelines would need to detail a practical and cost-effective approach to identify those at high risk of obesity-related complications (without imaging or biopsy studies) best placed for anti-platelet-based cancer chemoprotection. Non-invasive markers of fibrosis, e.g. fibrosis-4 score could be used to triage patients into more specific investigations.

Our study has several strengths, including being the first and largest real-world study performed in a Western population to assess the impact of anti-platelet therapy on protection against obesity-related cancers in people with NAFLD. The topic is highly clinically relevant given the rising rates of obesity in adults and children [2], and subsequently NAFLD, and the burden of hepatic and extrahepatic cancers in this group. Of note, the composite outcome of ‘obesity-related’ cancer used in this paper was chosen given the clinical relevance of this topic and strong evidence in the literature linking certain cancers with obesity. It does not translate that all the incident cancer cases reported here were directly related to obesity and instead will have occurred as a result of a complex interplay of metabolic risk, lifestyle factors and genetics.

We must acknowledge some limitations. Firstly, these are real-world data, and comparisons are not randomised nor controlled. Therefore, we cannot comment on causation. Second, resulting from data being extracted from electronic health records of an administrative database, there is potential for a lack of data completeness. For example, data may not be recorded by the HCO, such as the dose, or duration, of treatment, or recorded in free text that we are unable to extrapolate. We attempted to mitigate against challenges faced in determining treatment duration by ensuring that repeat coding for anti-platelet therapy was present. NAFLD may have resolved in some participants over the study time course. We were unable to identify which patients had experienced NAFLD resolution as such assessment would require serial biopsies or imaging data that was not available to us. Related, we could not analyse the impact of individual anti-platelets, aside from aspirin, on obesity-related cancer outcomes, as the sample size was too small. Moreover, as with any retrospective database study, despite thorough covariate adjustment through PSM at baseline, it is possible that minimal residual bias confounding remains. We attempted to reduce risk of unidentified residual confounding through calculation of *E*-values as a quantitative bias analysis to assist readers in the interpretation of the strength of our results [24]. The 1-year time lag may have introduced an element of immortality bias, but this was consistent between groups. In addition, a sensitivity analysis from the point of drug initiation was performed which further supports our findings. Individuals adhering to long-term medication may have higher socioeconomic status and healthier lifestyles which are better controlled for and evaluated through RCTs. We could not evaluate outcomes over a longer period of follow-up (e.g. 10 years) due to the loss of signal. Finally, although ICD-10 revision coding is a validated method for identifying disease outcomes, variability in diagnostic and coding practices might influence its accuracy. We used diagnostic codes that pertain to the diagnosis of ‘NAFLD’, despite the recent update in the nomenclature to metabolic dysfunction associated steatotic liver disease (MASLD) as there are significant gaps in metabolic data measured which is necessary to make a diagnosis of MASLD.

## Conclusions

In summary, our study highlights the novel potential of anti-platelet therapy, notably aspirin, in reducing the incidence of several hepatic and extra-hepatic obesity-related cancers, in individuals with NAFLD, a high-risk population. Randomised, controlled studies should explore their potential primary cancer prevention role.

## Supplementary Information

Additional File 1: Table S1. STROBE checklist. Table S2. ICD-10 codes used to exclude people with other aetiologies of chronic liver disease when creating the study cohorts. Table S3. ICD-10 codes of other obesity-related carcinomas. Table S4. Summary of outcomes of aspirin users stratified by age (people prescribed aspirin monotherapy vs non-users of any antiplatelets). Table S5. Summary of outcomes of aspirin users stratified by length of exposure (people prescribed aspirin monotherapy vs non-users of any antiplatelets). Fig. S1. Forest plot of all clinical outcomes at 5 years in aspirin users with MASLD, sub-stratified by age. Fig. S2. Forest plot of all clinical outcomes at 5 years in aspirin users with MASLD, sub-stratified by length of aspirin exposure.

## Data Availability

The data that support the findings of this study are available from TriNetX, LLC but third-party restrictions apply to the availability of these data. The data were used under license for this study with restrictions that do not allow for the data to be redistributed or made publicly available. However, for accredited researchers, the TriNetX data is available for licensing at TriNetX, LLC. To gain access to the data in the TriNetX research network, a request can be made to TriNetX (https://live.trinetx.com), but costs may be incurred, a data sharing agreement would be necessary, and no patient identifiable information can be obtained.

## Abbreviations

COX: Cyclooxygenase
HCC: Hepatocellular carcinoma
HCOs: Health care organisations
ICD-10: International Classification of Diseases 10th revision
MASLD: Metabolic dysfunction associated steatotic liver disease
NAFLD: Non-alcoholic fatty liver disease
PSM: Propensity score matched
RCT: Randomised controlled trial
SSMD: Strictly standardised mean difference
STROBE: Strengthening the Reporting of Observational Studies in Epidemiology
USPSTF: US Preventive Services Task force
