# Correction: The chemoprotective effect of anti-platelet agents on cancer incidence in people with non-alcoholic fatty liver disease (NAFLD): a retrospective cohort study

**DOI:** 10.1186/s12916-025-04033-x

**Published:** 2025-03-31

**Authors:** Matthew Anson, Jun Shang Poon, Alex E. Henney, David Riley, Gema H. Ibarbaru, Cyril Sieberhagen, Daniel J. Cuthbertson, Uazman Alam, Theresa Hydes

**Affiliations:** 1 Department of Cardiovascular and Metabolic Medicine, Institute of Life Course and Medical Sciences, University of Liverpool, Liverpool, UK; 2 Liverpool Centre for Cardiovascular Science, University of Liverpool, Liverpool, UK; 3 University Hospital Aintree, Liverpool University Hospitals NHS Foundation Trust, Liverpool, UK; 4 TriNetX LLC, Cambridge, MA USA

**Correction: BMC Med 22, 574 (2024)**

**https://doi.org/10.1186/s12916-024-03802-4**

The original article contained an error in the following sentence: “We adopted an active comparator new user design”. This is incorrect and should read “We adopted a new-user vs non-user design, where analysis was of new starters of antiplatelets comparted to non-exposed individuals.” The study methodology subsequently described in the original article reflects this and the incorrect initial description of the study design is because of simple human error.

Figure 1 contained a graphical error where the non-user group is clearly presented in red and described as “no antiplatelet group” within the legend, but the y axis indicates that they were users of antiplatelets (which they were not). Figure 1 has been revised to better reflect the study methodology.

**Fig. 1:**
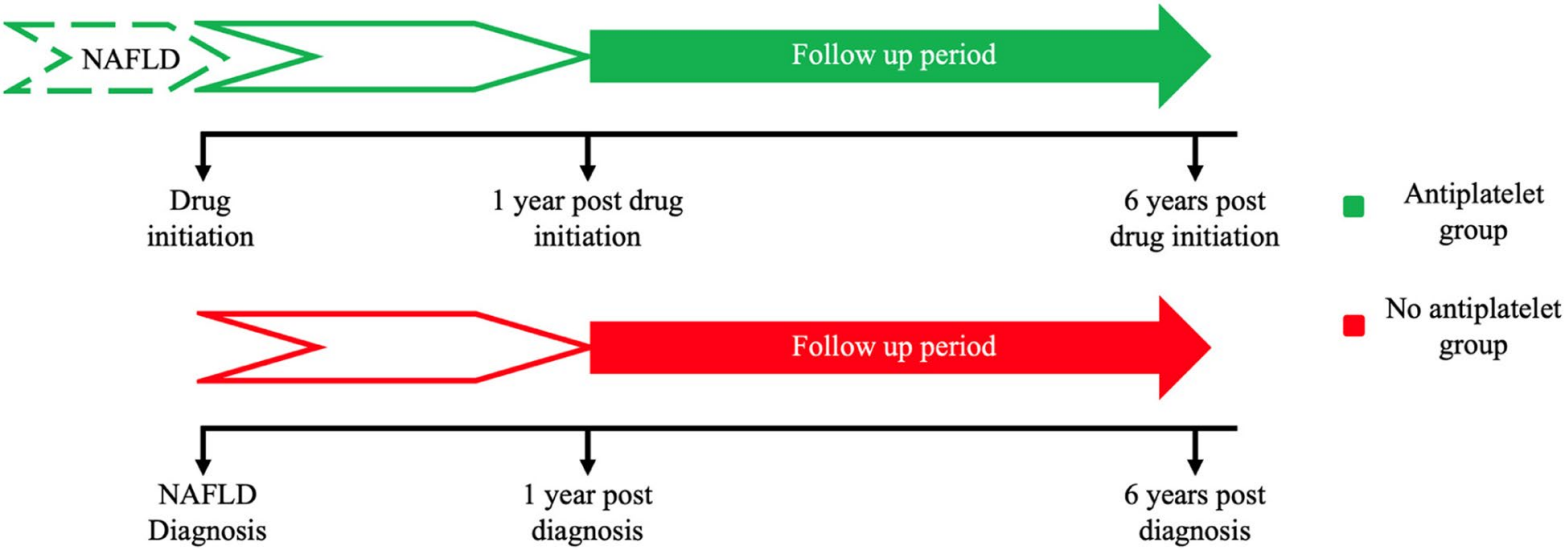
Timeline of included individuals for the main cohort analysis

These errors have no effect on the statistical significance of any values presented, nor did it affect other results reported in the paper.

We have provided pre-PSM characteristics in additional supplementary table (**table S6**), this is not a correction.

**Table S6:** Baseline patient demographics and characteristics **pre** propensity score matching.

**Table:** 

	**Anti-platelet use** **(n = 42,649)**	**No antiplatelet use (n = 1,044,035)**	**Strictly Standardised Mean Difference**
**Demographics**			
Age at index event (years)	62.8 ± 12.6	49.7 ± 16.3	0.899
Sex (Female) [%]	58	59	0.021
Race (White / Black or African American / Asian) [%]	71/9/4	63/7/5	0.163/0.095/0.042
**Anthropometrics**			
Body mass index (kg/m^2^)	33.4 ± 7.6	34.2 ± 8.1	0.101
**Comorbidities** [%]			
Type 2 diabetes	33.8	9.3	0.623
Ischaemic heart disease	20.2	1.7	0.621
History of any neoplasm	14.4	8.7	0.179
Cerebrovascular disease	6.7	0.7	0.321
Other peripheral vascular disease	3.3	0.4	0.218

We have provided further comment in the discussion, which is not a correction, but serves as added wider context: Comparing treatment versus no-treatment may introduce confounding by indication of antiplatelet use. Additionally, the control group was followed up one-year post-NAFLD diagnosis, whereas the exposed group may be more advanced in their diagnosis. Unfortunately, the length of NAFLD diagnosis is unavailable to us. Despite this, both these potential confounders may introduce a form of negative confounding, where individuals prescribed antiplatelets are inherently at greater risk of cardiovascular disease and malignancy, likely leading to an underestimation of the true protective effect of antiplatelets. Since these individuals may have had NAFLD for a longer period, they are expected to have worse health outcomes independent of antiplatelet use, further biasing our results toward the null. Therefore, our findings likely do not overestimate, but rather underestimate, the true effect of antiplatelets in this cohort.
