# Supplementary material for: The chemoprotective effect of anti-platelet agents on cancer incidence in people with non-alcoholic fatty liver disease (NAFLD): a retrospective cohort study
Source: BMC Med. 2024 Dec 3;22:574. doi: 10.1186/s12916-024-03802-4 (PMC11613771; doi:10.1186/s12916-024-03802-4)

**Additional Files 1**

**Additional File 1: Table S1.** STROBE checklist

|  | Item No | Recommendation | Page No |
| --- | --- | --- | --- |
| **Title and abstract** | 1 | (*a*) Indicate the study’s design with a commonly used term in the title or the abstract | 1, 2 |
|  |  | (*b*) Provide in the abstract an informative and balanced summary of what was done and what was found |  |
| Introduction | | | |
| Background/rationale | 2 | Explain the scientific background and rationale for the investigation being reported | 3 |
| Objectives | 3 | State specific objectives, including any prespecified hypotheses | 4 |
| Methods | | | |
| Study design | 4 | Present key elements of study design early in the paper | 4 |
| Setting | 5 | Describe the setting, locations, and relevant dates, including periods of recruitment, exposure, follow-up, and data collection | 4,5 |
| Participants | 6 | (*a*) Give the eligibility criteria, and the sources and methods of selection of participants. Describe methods of follow-up | 5 |
|  |  | (*b*) For matched studies, give matching criteria and number of exposed and unexposed |  |
| Variables | 7 | Clearly define all outcomes, exposures, predictors, potential confounders, and effect modifiers. Give diagnostic criteria, if applicable | 5 |
| Data sources/ measurement | 8* | For each variable of interest, give sources of data and details of methods of assessment (measurement). Describe comparability of assessment methods if there is more than one group | 5 |
| Bias | 9 | Describe any efforts to address potential sources of bias | 5 |
| Study size | 10 | Explain how the study size was arrived at | 5 |
| Quantitative variables | 11 | Explain how quantitative variables were handled in the analyses. If applicable, describe which groupings were chosen and why | 5 |
| Statistical methods | 12 | (*a*) Describe all statistical methods, including those used to control for confounding | 5 |
|  |  | (*b*) Describe any methods used to examine subgroups and interactions |  |
|  |  | (*c*) Explain how missing data were addressed |  |
|  |  | (*d*) If applicable, explain how loss to follow-up was addressed |  |
|  |  | (*e*) Describe any sensitivity analyses |  |
| Results | | |  |
| Participants | 13* | (a) Report numbers of individuals at each stage of study—eg numbers potentially eligible, examined for eligibility, confirmed eligible, included in the study, completing follow-up, and analysed | 5, 6 |
|  |  | (b) Give reasons for non-participation at each stage |  |
|  |  | (c) Consider use of a flow diagram |  |
| Descriptive data | 14* | (a) Give characteristics of study participants (eg demographic, clinical, social) and information on exposures and potential confounders | Table 1 |
|  |  | (b) Indicate number of participants with missing data for each variable of interest |  |
|  |  | (c) Summarise follow-up time (eg, average and total amount) |  |
| Outcome data | 15* | Report numbers of outcome events or summary measures over time | Table 2-Supplementary table 2 |

**Additional File 1: Table S2.** ICD-10 codes used to exclude people with other aetiologies of chronic liver disease when creating the study cohorts

| **Exclusion criteria** | **ICD-10 code** |
| --- | --- |
| Alcoholic liver disease | K70 |
| Alcoholic hepatitis | K70.1 |
| Alcoholic fibrosis and sclerosis of liver | K70.2 |
| Alcoholic cirrhosis of liver | K70.3 |
| Alcoholic hepatic failure | K70.4 |
| Alcoholic liver disease, unspecified | K70.9 |
| Alcohol induced acute pancreatitis | K85.2 |
| Alcohol induced chronic pancreatitis | K86.0 |
| Chronic viral hepatitis | B18 |
| Unspecified viral hepatitis | B19 |
| Disorders of copper metabolism | E83.0 |
| Disorders of iron metabolism | E83.1 |
| Disorders of plasma protein metabolism, not elsewhere classified | E88.0 |
| Budd-Chiari syndrome | I82.0 |
| Toxic liver disease | K71 |
| Chronic active hepatitis, not elsewhere classified | K73.2 |
| Chronic hepatitis, unspecified | K73.9 |
| Primary biliary cirrhosis | K74.3 |
| Secondary biliary cirrhosis | K74.4 |
| Biliary cirrhosis, unspecified | K74.5 |
| Autoimmune hepatitis | K75.4 |
| Hepatic veno-occlusive disease | K76.5 |
| Cholangitis | K83.0 |
| Alcohol induced pseudo-Cushing’s syndrome | E24.4 |
| Alcohol abuse | F10.1 |
| Alcohol dependence | F10.2 |
| Degeneration of nervous system due to alcohol | G31.2 |
| Alcoholic polyneuropathy | G62.1 |
| Alcoholic myopathy | G72.1 |
| Alcoholic cardiomyopathy | I42.6 |
| Alcoholic gastritis | K29.2 |
| Toxic effect of alcohol | T51 |
| Toxic effect of unspecified alcohol | T51.9 |

| **Outcome criteria** | **ICD-10 code** |
| --- | --- |
| Malignant neoplasm of oesophagus | C15 |
| Malignant neoplasm of stomach | C16 |
| Malignant neoplasm of colon | C18 |
| Malignant neoplasm of rectosigmoid junction | C19 |
| Malignant neoplasm of gallbladder | C23 |
| Malignant neoplasm of pancreas | C25 |
| Malignant neoplasm of breast | C50 |
| Malignant neoplasm of uterus, part unspecified | C55 |
| Malignant neoplasm of ovary | C56 |
| Malignant neoplasm of thyroid gland | C73 |
| Multiple myeloma | C90 |

**Additional File 1: Table S3.** ICD-10 codes of other obesity-related carcinomas

**Additional File 1: Table S4.** Summary of outcomes of aspirin users stratified by age (people prescribed aspirin monotherapy vs non-users of any antiplatelets)

|  | **> 60 years [aspirin] (n=40,248)** | | | | | **≤ 60 years [aspirin] (n=17,692)** | | | | |
| --- | --- | --- | --- | --- | --- | --- | --- | --- | --- | --- |
|  | **Sample size** | **Outcome (n)** | **HR (95% confidence interval)** | ***p* value** | **E value** | **Sample size** | **Outcome (n)** | **HR (95% confidence interval)** | ***p* value** | **E value** |
| ***All obesity-related cancers**** | | | | | | | | | | |
| Aspirin | 17,445 | 424 | **0.72 (0.63-0.82)** | **<0.001** | **1.82** | 8,252 | 93 | 0.78 (0.60-1.03) | 0.589 | 1.00 |
| No antiplatelets | 18,003 | 564 |  |  |  | 8,417 | 112 |  |  |  |
| ***Hepatocellular carcinoma*** | | | | | | | | | | |
| Aspirin | 19,888 | 41 | **0.41 (0.28-0.59)** | **<0.001** | **3.09** | 8,809 | 10^ϕ^ | 1.87 (0.64-5.47) | 0.246 | 1.00 |
| No antiplatelets | 19,957 | 95 |  |  |  | 8,834 | 10^ϕ^ |  |  |  |
| ***Colorectal carcinoma*** | | | | | | | | | | |
| Aspirin | 19,975 | 82 | **0.67 (0.51-0.90)** | **0.006** | **1.97** | 8,724 | 13 | 0.64 (0.32-1.30) | 0.212 | 1.00 |
| No antiplatelets | 19,701 | 114 |  |  |  | 8,764 | 29 |  |  |  |
| ***Pancreatic carcinoma*** | | | | | | | | | | |
| Aspirin | 19,978 | 55 | **0.70 (0.49-0.99)** | **0.040** | **1.88** | 8,820 | 10^ϕ^ | 0.94 (0.37-2.36) | 0.887 | 1.00 |
| No antiplatelets | 19,945 | 74 |  |  |  | 8,817 | 10^ϕ^ |  |  |  |
| ***Oesophageal carcinoma*** | | | | | | | | | | |
| Aspirin | 20,079 | 14 | 0.63 (0.32-1.23) | 0.172 | 1.00 | 8,839 | 10^ϕ^ | 0.47 (0.09-2.54) | 0.366 | 1.00 |
| No antiplatelets | 20,068 | 21 |  |  |  | 8,839 | 10^ϕ^ |  |  |  |
| ***Gastric carcinoma*** | | | | | | | | | | |
| Aspirin | 20,057 | 26 | **0.59 (0.36-0.97)** | **0.035** | **2.24** | 8,836 | 10^ϕ^ | 0.52 (0.17-1.54) | 0.229 | 1.00 |
| No antiplatelets | 20,058 | 41 |  |  |  | 8,835 | 10^ϕ^ |  |  |  |
| ***Gallbladder carcinoma*** | | | | | | | | | | |
| Aspirin | 20,103 | 10^ϕ^ | 1.18 (0.32-4.40) | 0.804 | 1.00 | 8,844 | 10^ϕ^ | 0.32 (0.03-3.03) | 0.290 | 1.00 |
| No antiplatelets | 20,104 | 10^ϕ^ |  |  |  | 8,846 | 10^ϕ^ |  |  |  |
| ***Ovarian carcinoma*** | | | | | | | | | | |
| Aspirin | 20,000 | 30 | 0.64 (0.40-1.01) | 0.054 | 1.00 | 8,806 | 12 | 0.80 (0.37-1.73) | 0.570 | 1.00 |
| No antiplatelets | 20,024 | 44 |  |  |  | 8,819 | 14 |  |  |  |
| ***Uterine carcinoma*** | | | | | | | | | | |
| Aspirin | 19,850 | 62 | 0.87 (0.62-1.24) | 0.447 | 1.00 | 8,774 | 11 | 0.49 (0.24-1.01) | 0.050 | 1.00 |
| No antiplatelets | 19,842 | 66 |  |  |  | 8,794 | 21 |  |  |  |
| ***Breast carcinoma*** | | | | | | | | | | |
| Aspirin | 18,963 | 145 | **0.74 (0.59-0.92)** | **0.005** | **1.77** | 8,650 | 34 | 0.88 (0.55-1.40) | 0.584 | 1.00 |
| No antiplatelets | 18,324 | 186 |  |  |  | 8,673 | 36 |  |  |  |
| ***Multiple myeloma*** | | | | | | | | | | |
| Aspirin | 19,958 | 32 | 1.00 (0.61-1.64) | 0.988 | 1.00 | 8,795 | 10^ϕ^ | 1.16 (0.31-4.31) | 0.828 | 1.00 |
| No antiplatelets | 20,052 | 30 |  |  |  | 8,836 | 10^ϕ^ |  |  |  |
| ***Thyroid carcinoma*** | | | | | | | | | | |
| Aspirin | 19,912 | 34 | 0.79 (0.50-1.25) | 0.317 | 1.00 | 8,765 | 16 | 1.06 (0.52-2.18) | 0.869 | 1.00 |
| No antiplatelets | 19,996 | 40 |  |  |  | 8,787 | 14 |  |  |  |

*Individuals were censored at the first coding of a constituent NAFLD-related malignancy composite outcome. The total number of individuals experiencing the composite outcome differ than that of the sum of the individual events because, to better ascertain the primary preventative effect of aspirin on all NAFLD-related malignancies, individuals with a history of any of the constituent events were excluded from analysis of the composite outcome.

^ϕ^ TriNetX implements several safeguards to minimize the risk of patient reidentification. To avoid the risk that a series of individual queries could identify small subsets of cohorts, when a query returns a patient count on an outcome where the patient count is ≤ 10 but greater than 0, the count is obfuscated to 10. The reported HR is calculated without this obfuscation present.

**Additional File 1: Table S5.** Summary of outcomes of aspirin users stratified by length of exposure (people prescribed aspirin monotherapy vs non-users of any antiplatelets).

|  | **≥ 3 years (n=28,262)** | | | | | **≥ 5 years (n=14,124)** | | | | |
| --- | --- | --- | --- | --- | --- | --- | --- | --- | --- | --- |
|  | **Sample size** | **Outcome (n)** | **HR (95% confidence interval)** | ***p* value** | **E value** | **Sample size** | **Outcome (n)** | **HR (95% confidence interval)** | ***p* value** | **E Value** |
| ***All obesity-related cancers**** | | | | | | | | | | |
| Aspirin | 12,426 | 237 | **0.68 (0.57-0.80)** | **<0.001** | **1.94** | 5,877 | 120 | **0.76 (0.60-0.97)** | **0.025** | **1.71** |
| No antiplatelets | 12,804 | 348 |  |  |  | 6,066 | 159 |  |  |  |
| ***Hepatocellular carcinoma*** | | | | | | | | | | |
| Aspirin | 14,009 | 22 | **0.52 (0.31-0.88)** | **0.012** | **2.52** | 7,008 | 13 | **0.52 (0.27-0.99)** | **0.049** | **2.52** |
| No antiplatelets | 14,056 | 41 |  |  |  | 7,030 | 25 |  |  |  |
| ***Colorectal carcinoma*** | | | | | | | | | | |
| Aspirin | 13,802 | 53 | 0.77 (0.54-1.11) | 0.157 | 1.00 | 6,888 | 24 | 0.66 (0.40-1.11) | 0.116 | 1.00 |
| No antiplatelets | 13,858 | 67 |  |  |  | 6,916 | 36 |  |  |  |
| ***Pancreatic carcinoma*** | | | | | | | | | | |
| Aspirin | 14,055 | 30 | **0.54 (0.35-0.85)** | **0.007** | **2.43** | 7,022 | 14 | **0.40 (0.22-0.75)** | **0.003** | **3.16** |
| No antiplatelets | 14,016 | 53 |  |  |  | 6,999 | 34 |  |  |  |
| ***Oesophageal carcinoma*** | | | | | | | | | | |
| Aspirin | 14,102 | 10^ϕ^ | 0.52 (0.22-1.21) | 0.119 | 1.00 | 7,039 | 10^ϕ^ | 0.37 (0.01-1.38) | 0.121 | 1.00 |
| No antiplatelets | 14,093 | 15 |  |  |  | 7,042 | 10^ϕ^ |  |  |  |
| ***Gastric carcinoma*** | | | | | | | | | | |
| Aspirin | 14,085 | 13 | 0.57 (0.29-1.13) | 0.104 | 1.00 | 7,030 | 10^ϕ^ | 0.89 (0.36-2.20) | 0.804 | 1.00 |
| No antiplatelets | 14,080 | 22 |  |  |  | 7,035 | 10^ϕ^ |  |  |  |
| ***Gallbladder carcinoma*** | | | | | | | | | | |
| Aspirin | 14,121 | 10^ϕ^ | 0.16 (0.02-1.33) | 0.052 | 1.00 | 7,058 | 0 | N/A | N/A | N/A |
| No antiplatelets | 14,118 | 10^ϕ^ |  |  |  | 7,057 | 10^ϕ^ |  |  |  |
| ***Ovarian carcinoma*** | | | | | | | | | | |
| Aspirin | 14,042 | 12 | 0.51 (0.25-1.02) | 0.053 | 1.00 | 7,016 | 10^ϕ^ | 0.69 (0.26-1.81) | 0.449 | 1.00 |
| No antiplatelets | 14,067 | 23 |  |  |  | 7,024 | 10^ϕ^ |  |  |  |
| ***Uterine carcinoma*** | | | | | | | | | | |
| Aspirin | 13,943 | 34 | 0.77 (0.49-1.21) | 0.252 | 1.00 | 6,959 | 13 | 0.72 (0.35-1.47) | 0.364 | 1.00 |
| No antiplatelets | 13,957 | 43 |  |  |  | 6,973 | 18 |  |  |  |
| ***Breast carcinoma*** | | | | | | | | | | |
| Aspirin | 13,367 | 85 | 0.76 (0.57-1.01) | 0.054 | 1.00 | 6,605 | 42 | 0.84 (0.56-1.26) | 0.390 | 1.00 |
| No antiplatelets | 13,611 | 111 |  |  |  | 6,788 | 51 |  |  |  |
| ***Multiple myeloma*** | | | | | | | | | | |
| Aspirin | 14,029 | 14 | 0.66 (0.33-1.29) | 0.217 | 1.00 | 7,018 | 10^ϕ^ | 1.13 (0.43-2.92) | 0.808 | 1.00 |
| No antiplatelets | 14,091 | 21 |  |  |  | 7,041 | 10^ϕ^ |  |  |  |
| ***Thyroid carcinoma*** | | | | | | | | | | |
| Aspirin | 13,968 | 25 | 0.91 (0.53-1.57) | 0.734 | 1.00 | 6,974 | 13 | 1.01 (0.47-2.17) | 0.990 | 1.00 |
| No antiplatelets | 14,047 | 27 |  |  |  | 7,017 | 13 |  |  |  |

*Individuals were censored at the first coding of a constituent NAFLD-related malignancy composite outcome. The total number of individuals experiencing the composite outcome differ than that of the sum of the individual events because, to better ascertain the primary preventative effect of aspirin on all NAFLD-related malignancies, individuals with a history of any of the constituent events were excluded from analysis of the composite outcome.

^ϕ^ TriNetX implements several safeguards to minimize the risk of patient reidentification. To avoid the risk that a series of individual queries could identify small subsets of cohorts, when a query returns a patient count on an outcome where the patient count is ≤ 10 but greater than 0, the count is obfuscated to 10. The reported HR is calculated without this obfuscation present.

**Additional File 1: Figure S1.** Forest plot of all clinical outcomes at 5-years in aspirin users with MASLD, sub stratified by age


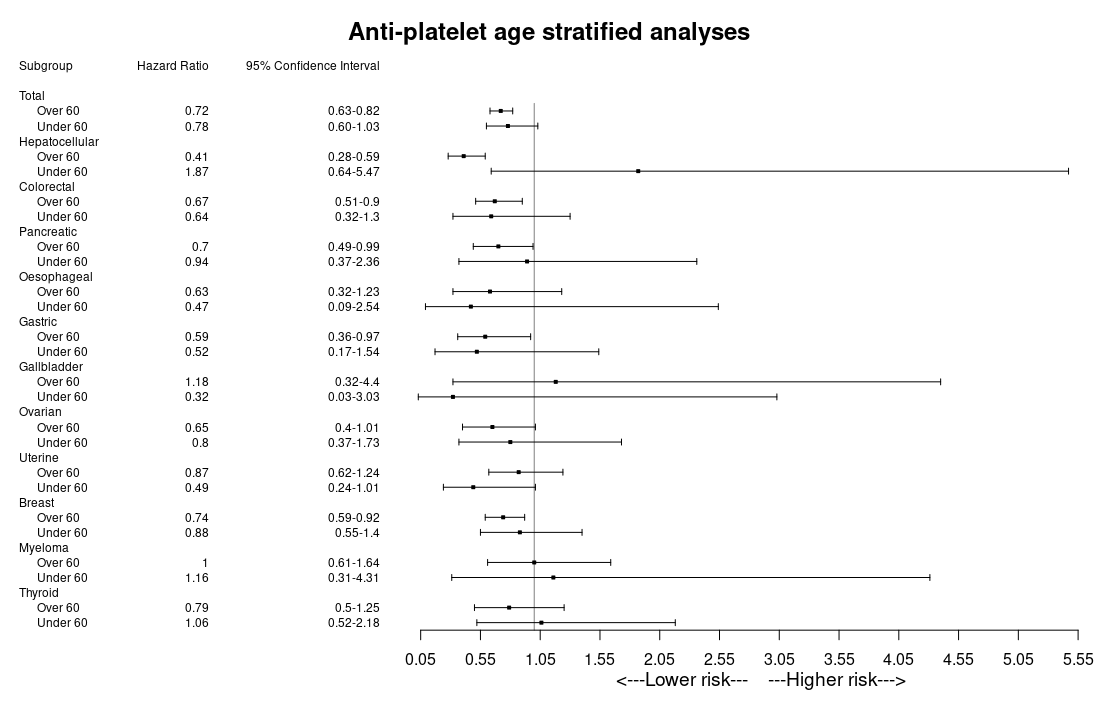


**Additional File 1: Figure S2.** Forest plot of all clinical outcomes at 5-years in aspirin users with MASLD, sub stratified by length of aspirin exposure


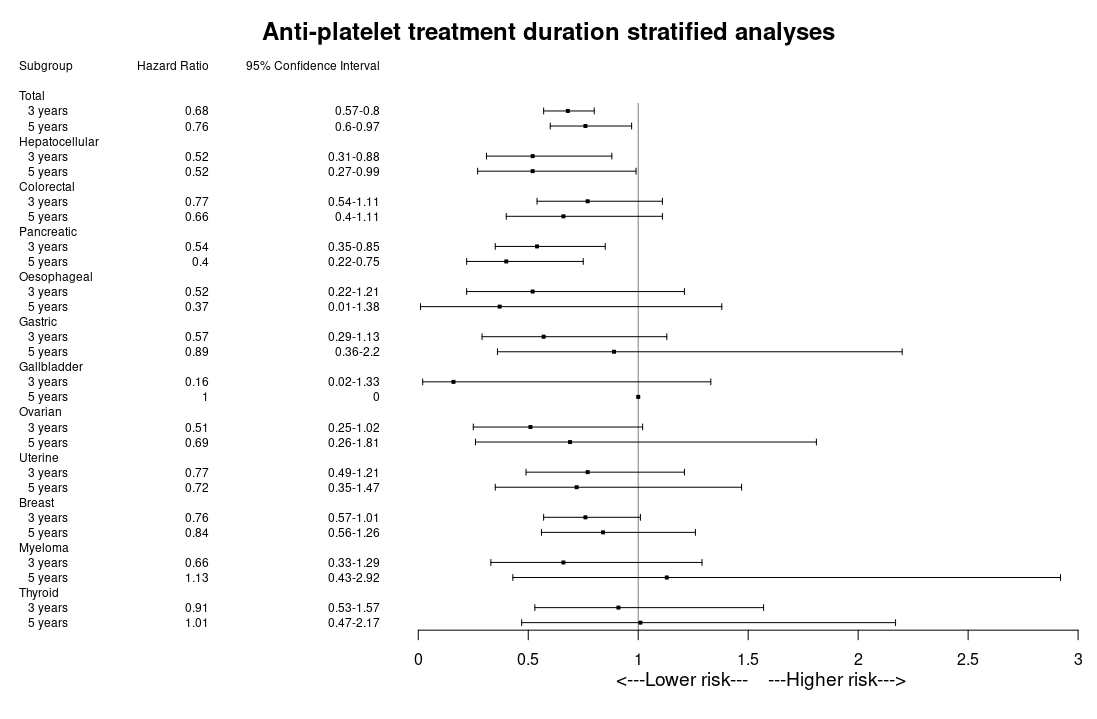

Supplement: Supplementary file 1 — Additional File 1: Table S1. STROBE checklist. Table S2. ICD-10 codes used to exclude people with other aetiologies of chronic liver disease when creating the study cohorts. Table S3. ICD-10 codes of other obesity-related carcinomas. Table S4. Summary of outcomes of aspirin users stratified by age (people prescribed aspirin monotherapy vs non-users of any antiplatelets). Table S5. Summary of outcomes of aspirin users stratified by length of exposure (people prescribed aspirin monotherapy vs non-users of any antiplatelets). Fig. S1. Forest plot of all clinical outcomes at 5 years in aspirin users with MASLD, sub-stratified by age. Fig. S2. Forest plot of all clinical outcomes at 5 years in aspirin users with MASLD, sub-stratified by length of aspirin exposure. [file 12916_2024_3802_MOESM1_ESM.docx]
